# Supplementary figures and images for: Heat‐Induced Secondary Dormancy Contributes to Local Adaptation in Arabidopsis thaliana
Source: Mol Ecol. 2025 Aug 26;34(19):e70086. doi: 10.1111/mec.70086 (PMC12456118; doi:10.1111/mec.70086)

A

common common unique

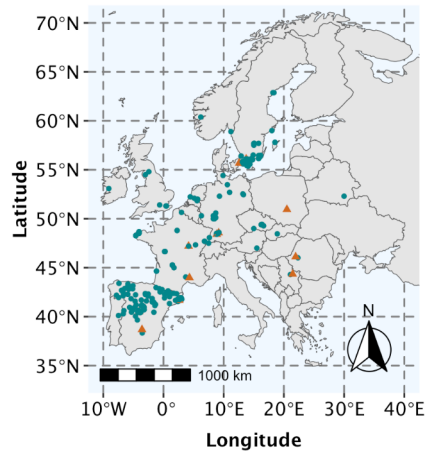

B

common common unique

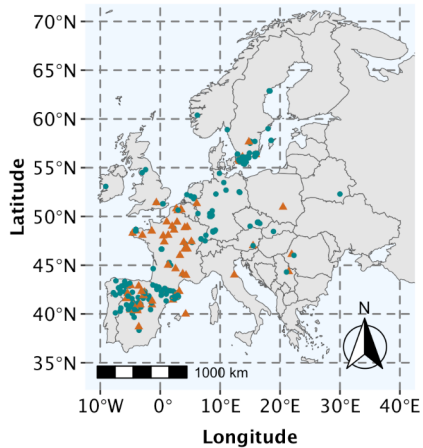

C

common common unique

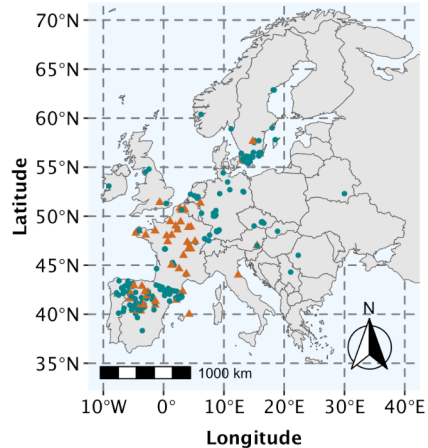

Supplement: Supplementary file 1 — Figure S1: Geographic origin of European Arabidopsis thaliana accessions used in this study. [file MEC-34-e70086-s002.pdf]

A

Germination rate

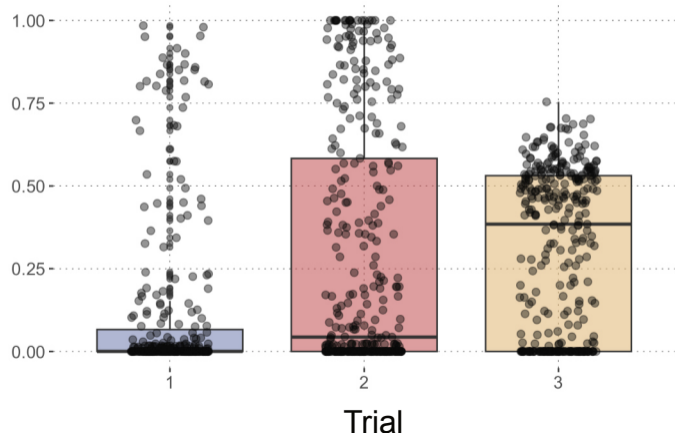

B

Residual

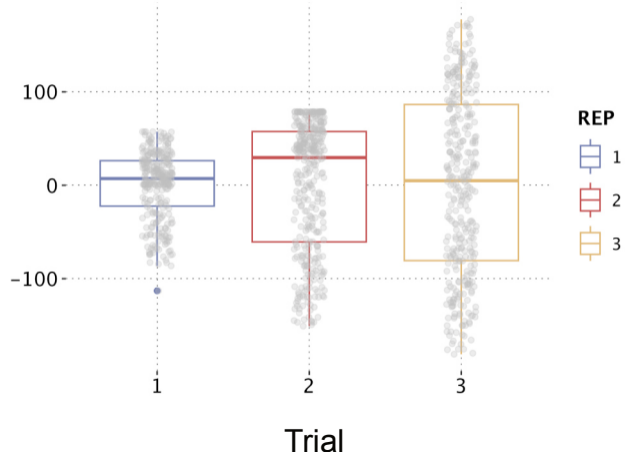

Supplement: Supplementary file 2 — Figure S2: Temporal dynamics of heat‐induced secondary dormancy. [file MEC-34-e70086-s005.pdf]

A

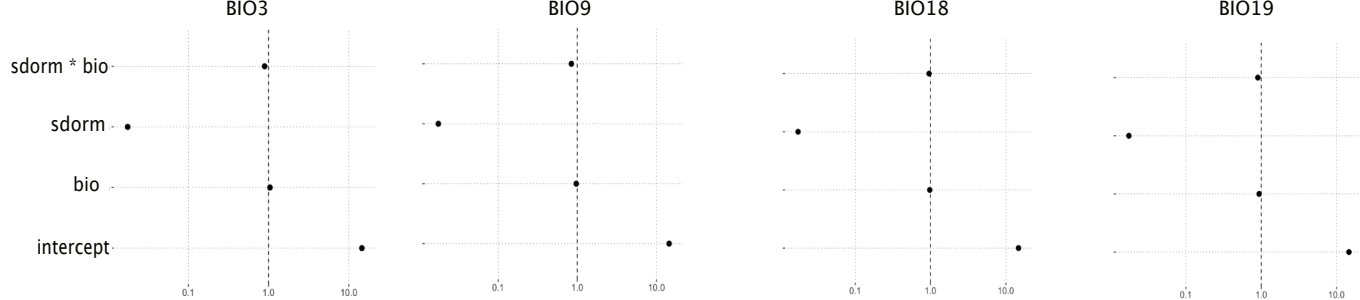

B

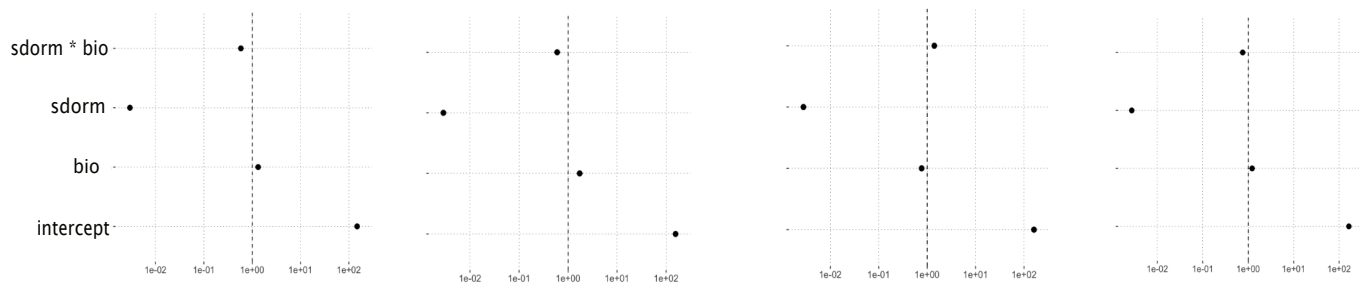

C

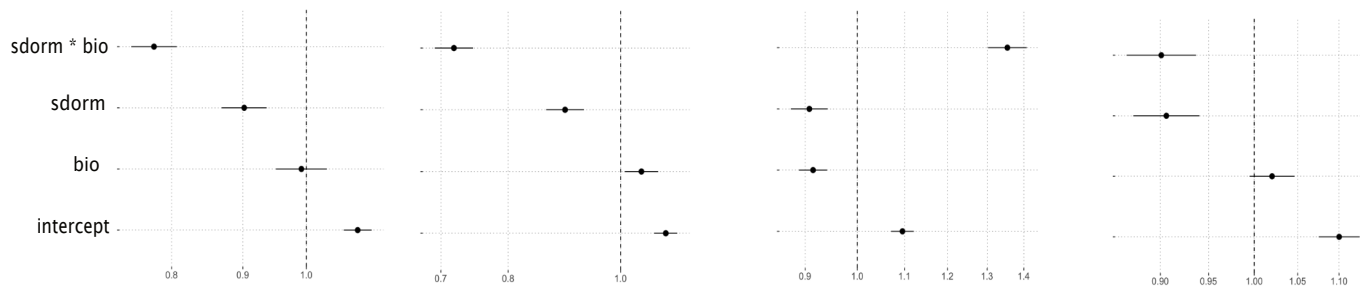

Supplement: Supplementary file 3 — Figure S3: Bioclimatic variables as predictors of heat‐induced secondary dormancy. [file MEC-34-e70086-s001.pdf]

A

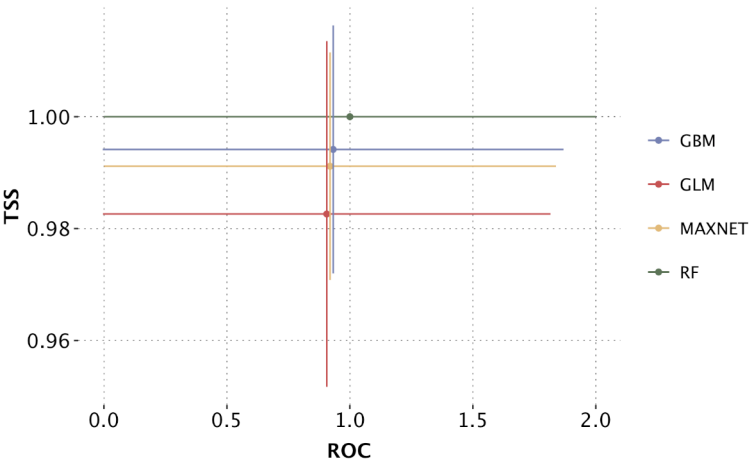

B

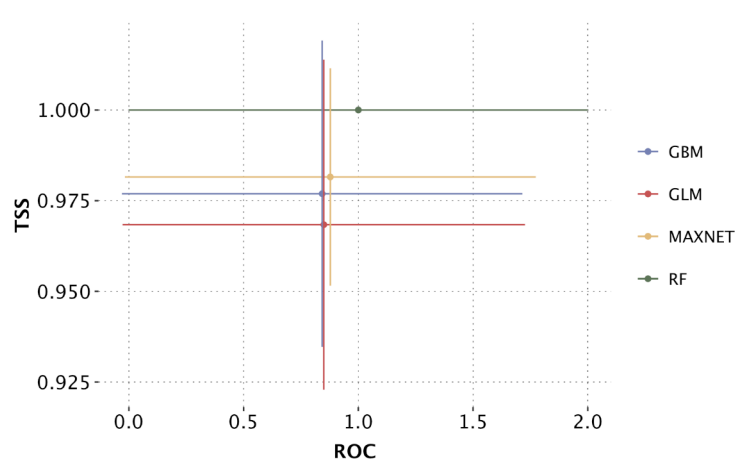

Supplement: Supplementary file 4 — Figure S4: Performance comparison of modelling approaches using ROC and TSS metrics in predicting heat‐induced secondary dormancy ecological niche. [file MEC-34-e70086-s006.pdf]

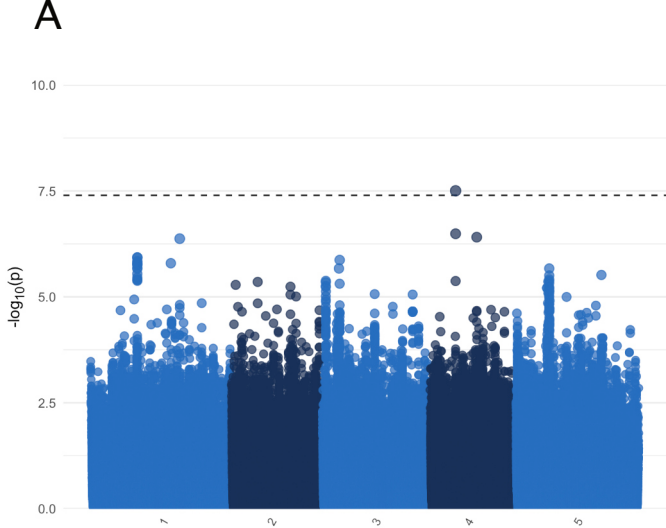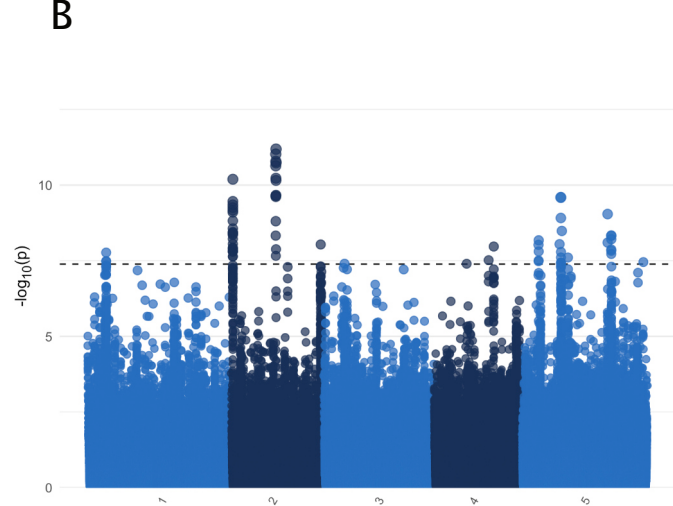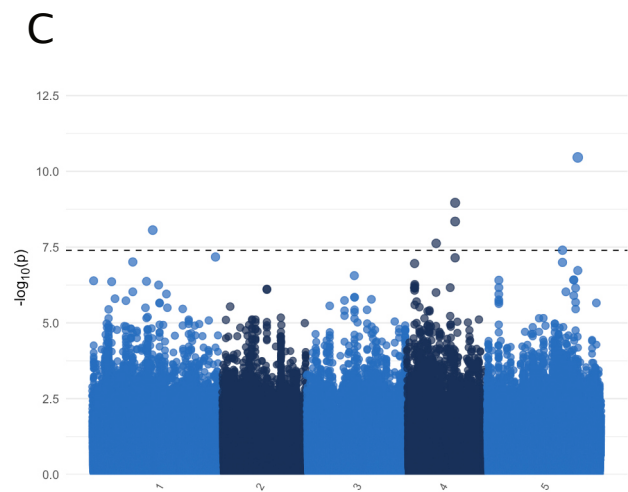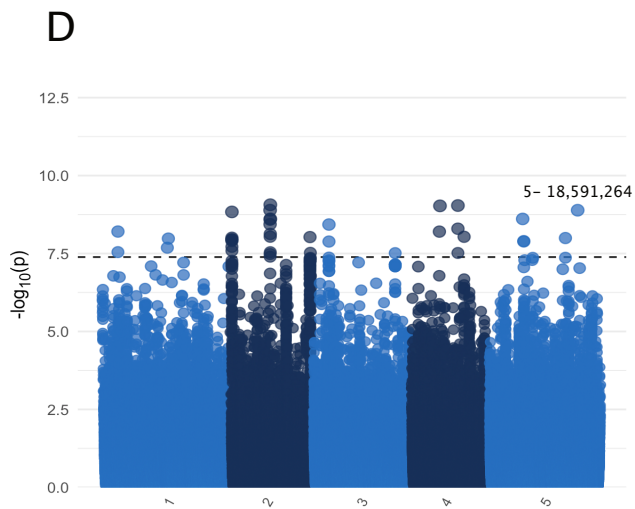

Supplement: Supplementary file 6 — Figure S6: Manhattan plot displaying the association of 1.2M SNP markers with primary dormancy across trials. [file MEC-34-e70086-s003.pdf]

A

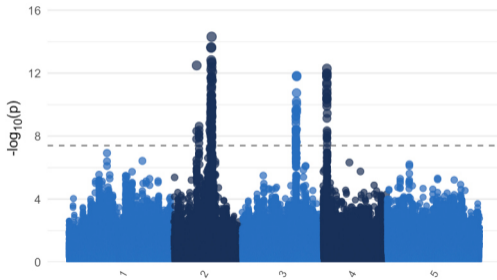

B

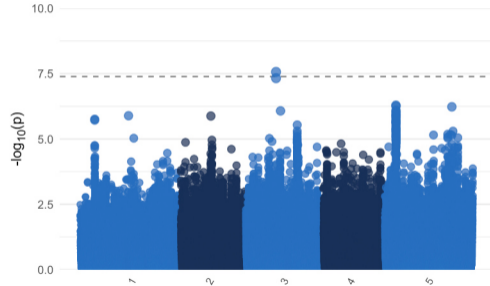

C

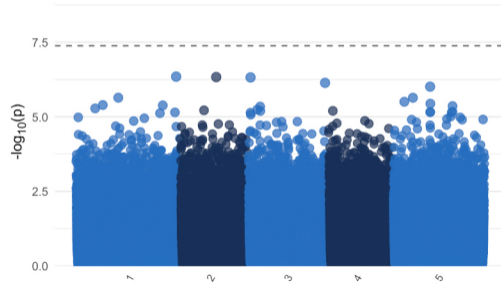

Supplement: Supplementary file 7 — Figure S7: Manhattan plot displaying the association of 1.2M SNP markers with heat‐induced secondary dormancy across trials. [file MEC-34-e70086-s008.pdf]
