# Supplementary material for: Heat‐Induced Secondary Dormancy Contributes to Local Adaptation in Arabidopsis thaliana
Source: Mol Ecol. 2025 Aug 26;34(19):e70086. doi: 10.1111/mec.70086 (PMC12456118; doi:10.1111/mec.70086)
Supplement: Supplementary file 5 — Figure S5: Importance of predictor variables in the species distribution model for strong and weak heat‐induced secondary dormancy ecotypes. [file MEC-34-e70086-s007.pdf]

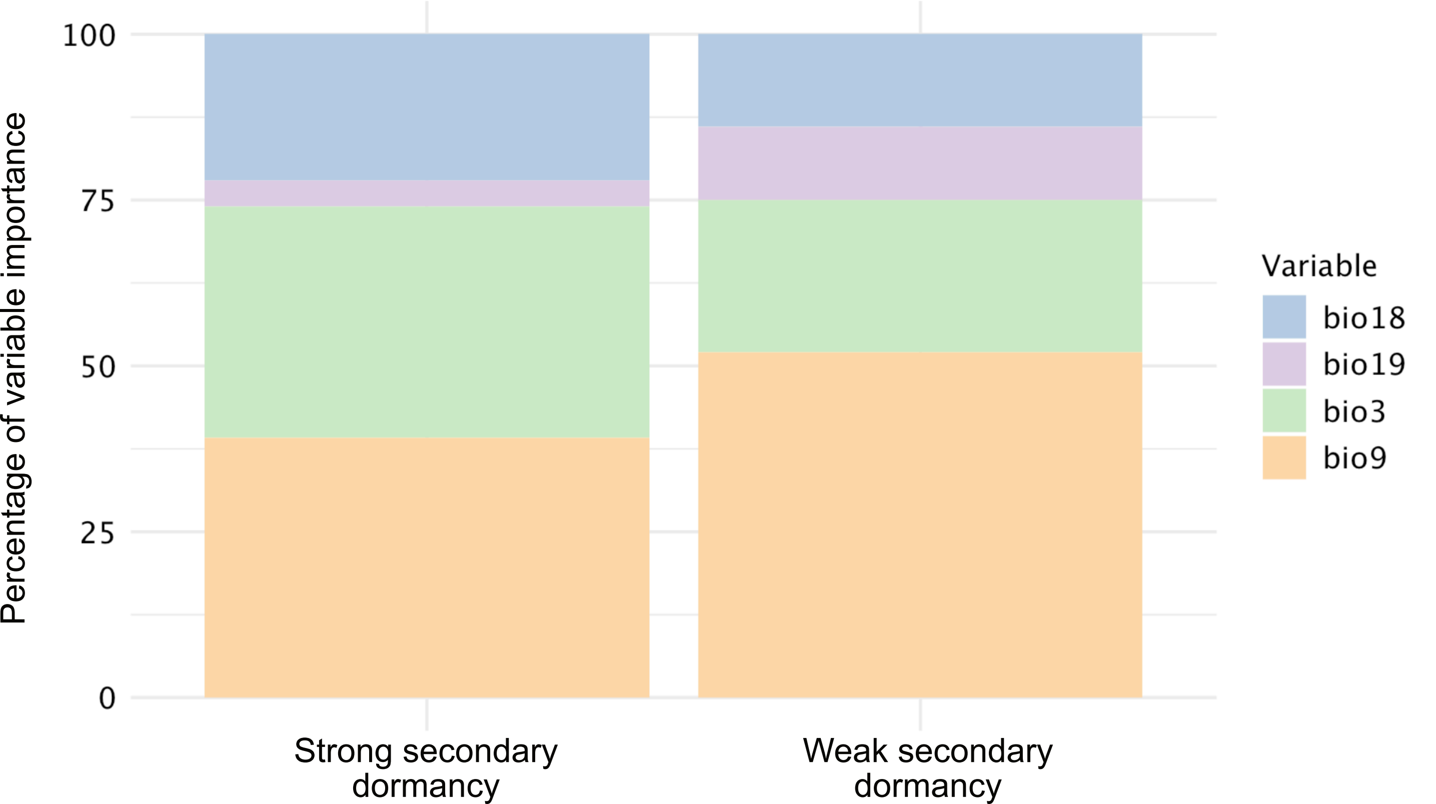

Percentage of variable importance
100
75
50
25
0
Strong secondary dormancy
Weak secondary dormancy
Variable
bio18
bio19
bio3
bio9
