# Supplementary material for: Heat‐Induced Secondary Dormancy Contributes to Local Adaptation in Arabidopsis thaliana
Source: Mol Ecol. 2025 Aug 26;34(19):e70086. doi: 10.1111/mec.70086 (PMC12456118; doi:10.1111/mec.70086)
Supplement: Supplementary file 8 — Table S1: Information of 361 studied accessions. Table S2: Spearman correlation coefficients for heat‐induced secondary dormancy across three trials. Table S3: Spearman correlation of residual primary dormancy and heat‐induced secondary dormancy of three trials. Table S4: Descriptive statistics of germination rates under heat‐induced secondary dormancy treatment across low‐ (< 50°) and high‐latitude (≥ 50°) regions in three trials. Table S5: Regression results of heat‐induced secondary dormancy with four bioclimatic variables as predictors of genetic variation in germination after three treatments, across three trials. Table S6: Genome‐wide association results of primary dormancy across three trials and shared genome‐wide association peaks across three experimental trials. Table S7: Genome‐wide association results of heat‐induced secondary dormancy across three trials and shared genome‐wide association peaks across three experimental trials. Table S8: Definitions of bioclimatic (BIO) variables used in the analysis and interpretation of their values. [file MEC-34-e70086-s004.docx]

Tables

Table S1. Information of 361 studied accessions. Table records Genotype ID (GenotypeID) according to ENA database and Genotype Name (GenotypeName) accordingly. Geographical origin (origin) and precise latitude and longitude of the genotype are recorded, together with extra information such as sequencer, collector, and CSS accession ID (AccessionID).

Attached as a separate file.

Table S2. Spearman correlation coefficients for heat-induced secondary dormancy across three trials. The Spearman correlation values quantifying the consistency of heat-induced secondary dormancy measurements across three independent trials. The secondary dormancy treatment represents germination rates following a 3-day stratification at 4°C to release dormancy, followed by a 4-day treatment at 37°C. The germination test was conducted under long-day conditions at 20°C, and germination rates were recorded after 7 days. All germination tests were conducted under long-day conditions at 20°C, and germination rates were recorded after 7 days. Trial 1 included 295 genotypes and was conducted in May 2022; Trial 2 comprised the full set of 361 genotypes and was conducted in December 2022; Trial 3 included 344 genotypes and was conducted in December 2023.

|  | rho | p value |
| --- | --- | --- |
| Trial 1 vs Trial 2 | 0.494 | < 2.2e-16 |
| Trial 2 vs Trial 3 | 0.409 | 3.661e-12 |
| Trial 1 vs Trail 3 | 0.350 | 4.393e-09 |

Table S3. Spearman correlation of residual primary dormancy and heat-induced secondary dormancy of three trials. Secondary dormancy treatment represents germination rates following a 3-day stratification at 4°C to release dormancy, followed by a 4-day treatment at 37°C. The primary dormancy treatment tested germination rates without any pre-treatment. All germination tests were conducted under long-day conditions at 20°C, and germination rates were recorded after 7 days. Trial 1 is a set of 295 samples, Trial 2 has the complete set of 361 samples, and Trial 3 is a set of 344 samples. Trial 1 was performed in May 2022, Trial 2 in December 2022, and Trial 3 in December 2023.

|  | rho | p value |
| --- | --- | --- |
| Trial 1 | 0.427 | < 1.6e-14 |
| Trial 2 | 0.204 | < 8.98e-05 |
| Trial 3 | 0.172 | = 0.00135 |

Table S4. Descriptive statistics of germination rates under heat-induced secondary dormancy treatment across low- (<50°) and high-latitude (≥50°) regions in three trials. We used 50^o^N as a threshold to separate northern and southern populations based on our assumptions of the biogeographical transitions in Central and Northern Europe. Secondary dormancy treatment represents germination rates following a 3-day stratification at 4°C to release dormancy, followed by a 4-day treatment at 37°C. All germination tests were conducted under long-day conditions at 20°C, and germination rates were recorded after 7 days. Trial 1 is a set of 295 samples, Trial 2 has the complete set of 361 samples, and Trial 3 is a set of 344 samples. Trial 1 was performed in May 2022, Trial 2 in December 2022, and Trial 3 in December 2023.

|  | Low latitude | High latitude |
| --- | --- | --- |
| Trial 1 | mean = 0.0511, var = 0.0278 | mean = 0.1808, var = 0.0826 |
| Trial 2 | mean = 0.1999, var = 0.1012 | mean = 0.4193, var = 0.152 |
| Trial 3 | mean = 0.2546, var = 0.0611 | mean = 0.3871, var = 0.0502 |

Table S5. Regression results of heat-induced secondary dormancy with four bioclimatic variables as predictors of genetic variation in germination after three treatments, across three trials. The results of regression models that assess the influence of four bioclimatic variables on secondary dormancy and its genetic variation. The models employed a binomial likelihood with a logit link function. The bioclimatic variables included BIO3 (isothermality), BIO9 (mean temperature of the driest quarter), BIO18 (mean precipitation of the warmest quarter), and BIO19 (mean precipitation of the coldest quarter). The analysis was performed across three treatments: primary dormancy (pdorm), secondary dormancy (sdorm), and control. Secondary dormancy was induced by a 4-day 37°C treatment following a 3-day stratification at 4°C to release primary dormancy. Primary dormancy was tested without any pre-treatment, whereas control seeds were stratified at 4°C for 3 days before germination testing. The sdorm value used in this model represents secondary dormancy corrected for primary dormancy, calculated as the residual of the regression of secondary dormancy on primary dormancy. All germination tests were conducted under long-day conditions at 20°C, and germination rates were recorded after 7 days. Trial 1 is a set of 295 samples, Trial 2 has the complete set of 361 samples, and Trial 3 is a set of 344 samples. Trial 1 was performed in May 2022, Trial 2 in December 2022, and Trial 3 in December 2023. (A), (B), and (C) represent models with the control group as the baseline for Trial 1, Trial 2, and Trial 3, respectively; (D), (E), and (F) represent models in whichprimary dormancy is used as the baseline, for Trial 1, Trial 2, and Trial 3, respectively.

Attached as a separate file.

Table S6. Genome-wide association results of primary dormancy across three trials and shared genome-wide association peaks across three experimental trials. The primary dormancy treatment tested germination rates without any pre-treatment. All germination tests were conducted under long-day conditions at 20°C, and germination rates were recorded after 7 days. (A) Trial 1 is a set of 295 samples, (B) Trial 2 is the complete set of 361 samples, and (C) Trial 3 is a set of 344 samples. Trial 1 was performed in May 2022, Trial 2 in December 2022, and Trial 3 in December 2023.  (D) Shared genome-wide association peaks of primary dormancy across all experimental trials, computed by Fisher’s combined probability test.

Attached as a separate file.

Table S7. Genome-wide association results of heat-induced secondary dormancy across three trials and shared genome-wide association peaks across three experimental trials. The secondary dormancy represents germination rates following a 3-day stratification at 4°C to release dormancy, followed by a 4-day treatment at 37°C. All germination test s were conducted under long-day conditions at 20°C, and germination rates were recorded after 7 days. (A) Trial 1 is a set of 295 samples, (B) Trial 2 is the complete set of 361 samples, and (C) Trial 3 is a set of 344 samples. Trial 1 was performed in May 2022, Trial 2 in December 2022, and Trial 3 in December 2023.  (D) Shared genome-wide association peaks of heat-induced secondary dormancy across all experimental trials, computed by Fisher’s combined probability test.

Attached as a separate file.

Table S8. Definitions of bioclimatic (BIO) variables used in the analysis and interpretation of their values. Descriptions of the nineteen bioclimatic (BIO) variables included in the generalized linear mixed model (GLMM). The ecological meaning of each BIO variable is provided, along with guidance on interpreting their values—particularly clarifying conditions represented by low and high values.

| **Bioclimatic variables** | **Definition** | **Intepretation** |
| --- | --- | --- |
| BIO1  Annual Mean Temperature | Mean of monthly temperature averages | High: warmer overall climate; Low: cooler year-round |
| BIO2  Mean Diurnal Range | Mean of monthly (max temp - min temp) | High: large day-night temperature shifts |
| BIO3  Isothermality | = (BIO2 / BIO7) × 100 | High: more uniform temp across year (diurnal ≈ seasonal); Low: high seasonal contrast relative to daily variation |
| BIO4  Temperature Seasonality | Standard deviation of temperature × 100 | High: large seasonal variability; Low: stable seasonal temps |
| BIO5  Max Temperature of Warmest Month | Highest monthly max temperature | High: hot extremes in summer |
| BIO6  Min Temperature of Coldest Month | Lowest monthly min temperature | Low: cold winters |
| BIO7  Temperature Annual Range | = BIO5 - BIO6 | High: strong summer–winter contrast; Low: mild year-round |
| BIO8  Mean Temperature of Wettest Quarter | Average temp during 3-month wettest period | Ecologically varies; important for timing of growth or dormancy |
| BIO9  Mean Temperature of Driest Quarter | Average temp during 3-month driest period | High: warm/dry periods; Low: cold/dry stress |
| BIO10  Mean Temp of Warmest Quarter | Average temp during warmest 3 months | High: hot growing seasons |
| BIO11  Mean Temp of Coldest Quarter | Average temp during coldest 3 months | Low: cold stress potential |
| BIO12  Annual Precipitation | Total yearly precipitation | High: wet climates; Low: arid regions |
| BIO13  Precipitation of Wettest Month | Total precipitation in the wettest month | High: strong rainy season |
| BIO14  Precipitation of Driest Month | Total precipitation in driest month | Low: drought severity |
| BIO15  Precipitation Seasonality | Coefficient of variation of monthly precipitation | High: unpredictable rain patterns |
| BIO16  Precipitation of Wettest Quarter | Precipitation total in wettest 3-month period | High: monsoon-heavy systems |
| BIO17  Precipitation of Driest Quarter | Precipitation total in driest 3-month period | Low: drought-prone periods |
| BIO18  Precipitation of Warmest Quarter | Precipitation in the warmest 3 months | Low: hot/dry stress; High: wet growing season |
| BIO19  Precipitation of Coldest Quarter | Precipitation in coldest 3 months | Low: potential for overwintering stress |
